# Twenty-five-year research progress in hookworm excretory/secretory products

**DOI:** 10.1186/s13071-020-04010-8

**Published:** 2020-03-14

**Authors:** Asmaa M. I. Abuzeid, Xue Zhou, Yue Huang, Guoqing Li

**Affiliations:** Guangdong Provincial Zoonosis Prevention and Control Key Laboratory, College of Veterinary Medicine, South China Agricultural University, Guangzhou, 510642 China

**Keywords:** Hookworm, ES products, Host-parasite interactions, Vaccine, Therapy

## Abstract

Hookworm infection is a major public health problem that threatens about 500 million people throughout tropical areas of the world. Adult hookworms survive for many years in the host intestine, where they suck blood, causing iron deficiency anemia and malnutrition. Numerous molecules, named excretory/secretory (ES) products, are secreted by hookworm adults and/or larvae to aid in parasite survival and pathobiology. Although the molecular cloning and characterization of hookworm ES products began 25 years ago, the biological role and molecular nature of many of them are still unclear. Hookworm ES products, with distinct structures and functions, have been linked to many essential events in the disease pathogenesis. These events include host invasion and tissue migration, parasite nourishment and reproduction, and immune modulation. Several of these products represent promising vaccine targets for controlling hookworm disease and therapeutic targets for many inflammatory diseases. This review aims to summarize our present knowledge about hookworm ES products, including their role in parasite biology, host-parasite interactions, and as vaccine and pharmaceutical targets and to identify research gaps and future research directions in this field.
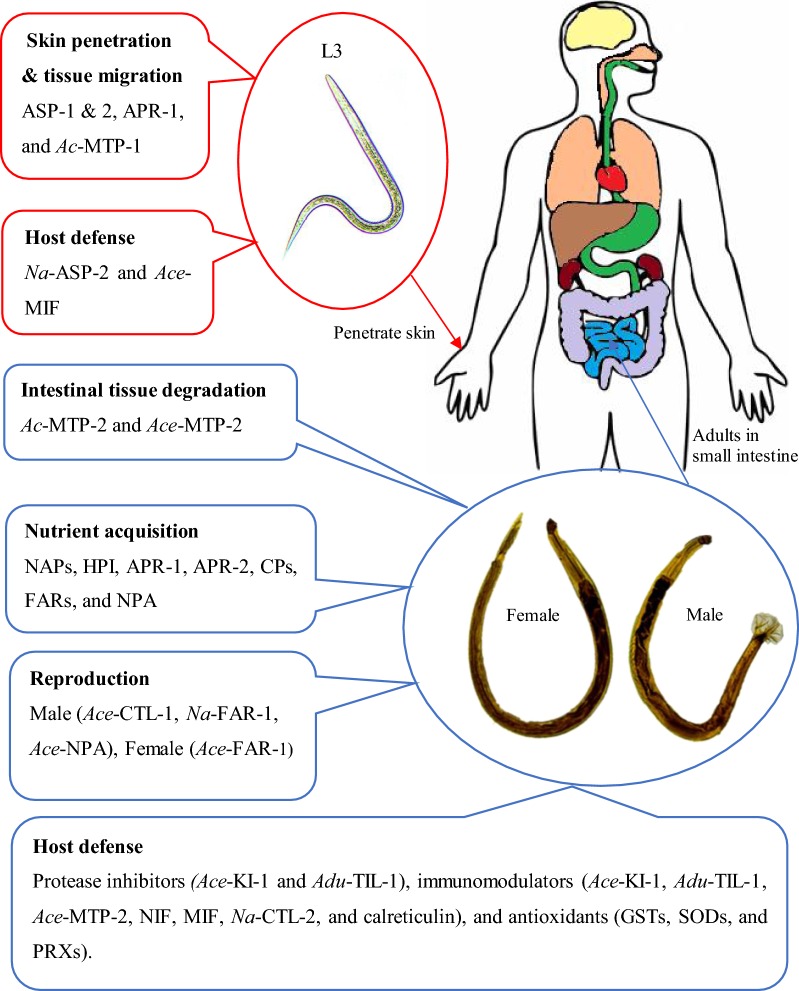

## Background

Hookworms are soil-transmitted intestinal nematodes that infect nearly 500 million people in tropical regions of South America, Africa and Asia [1]. These blood-feeding nematodes cause one of the most debilitating neglected tropical disease called hookworm disease. This disease resulted in four million disability-adjusted life years (DALYs) and up to USD139 billion annual economic productivity losses [2]. Most human hookworm infections are caused by *Necator americanus*, *Ancylostoma duodenale* and *Ancylostoma ceylanicum*. Hookworm-associated clinical symptoms, such as abdominal pain, diarrhea, and protein malnutrition, commonly occur. However, the principal clinical symptom of hookworm disease is iron deficiency anemia (IDA) because of blood losses [3, 4]. Chronic IDA affects all ages, but it is principally deleterious to persons with low iron stores (children and women of childbearing age). Sequelae of hookworm-associated chronic IDA include impaired physical and cognitive developments in children and raised perinatal maternal/infant mortalities in pregnant women [5].

Helminth key molecules comprise those present at the parasite external surface and the excretory/secretory (ES) products [6]. The ES products are a mixture of proteins, lipids and carbohydrates that are secreted from the parasite oral orifice or outer surfaces and represent the main boundary between the helminth parasites and their hosts [7]. Hookworm ES products contain a wide range of structurally and functionally distinct molecules, mostly proteins. These molecules react with host proteins and act major functions in parasite survival and development and host-parasite relationships. Notably, these molecules are crucial for the penetration of the host, tissue migration, nourishment, reproduction, and evasion of host immunity [7]. These molecules help the parasite to survive and evade the host immunological response by inhibiting the inflammatory reaction, encouraging effector cells apoptosis, and skewing the immune response phenotype [8]. Many hookworm ES products have proven as effective hookworm vaccine candidates [9] and therapeutic targets against inflammatory and allergic diseases [10].

Because of their importance in hookworm disease pathogenesis and control, hookworm ES products have been a target for intensive study for over two decades. Molecular cloning and characterization of hookworm ES proteins began with the work of Moyle et al. [11], who isolated integrin-blocking protein called neutrophil inhibitory factor (NIF) from the canine hookworm, *Ancylostoma caninum*. Progress in molecular biology since then has promoted the identification of several novel molecules from hookworm ES products. However, the function of many ES molecules is still unknown. Nearly two decades ago, Loukas and Prociv [8] reviewed hookworm molecules with an immunological impact. Then, Brooker et al. [5] provided a comprehensive list of hookworm molecules but with a brief description. Later, Pearson et al. [7] discussed the role of some ES proteins in the disease molecular mechanism. Molecular biological techniques have advanced over this period and thus have identified many new molecules. This review aims to summarize our knowledge about hookworm ES products, including their functions in host-parasite interactions and as a therapeutic target, and to identify research gaps and future research directions in this field.

## *Ancylostoma*-secretory proteins

**Fig. 1:**
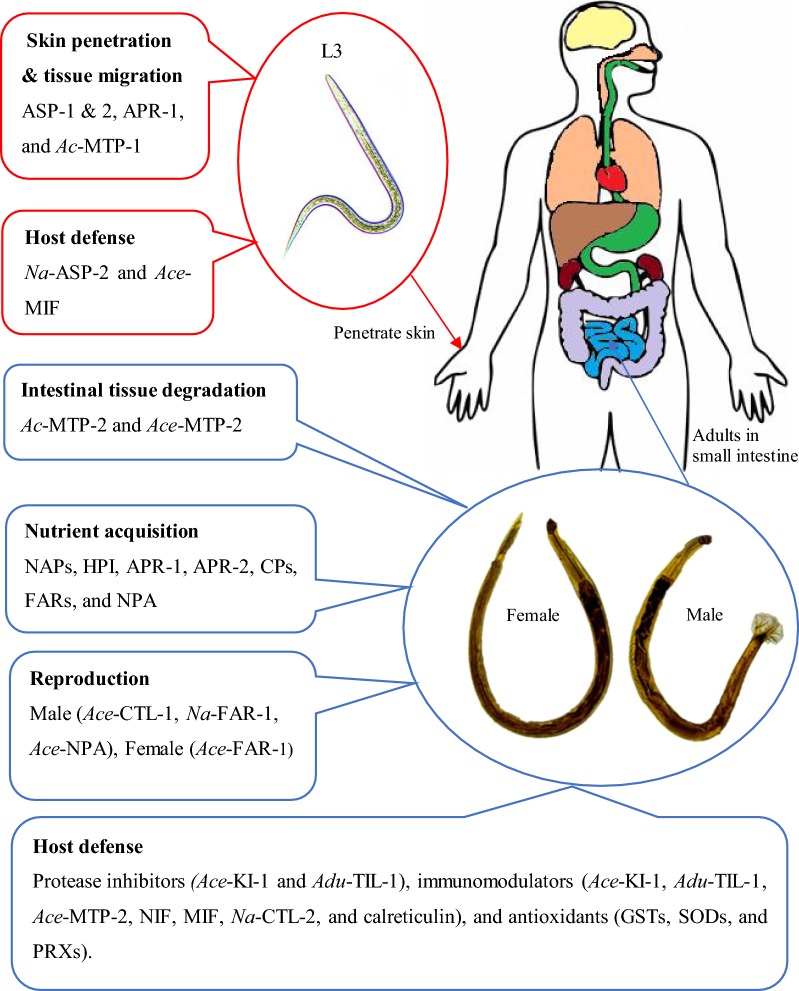
The role of hookworm excretory-secretory (ES) products in parasite biology and host-parasite interactions. Hookworm ES products are classified into molecules secreted from infective larvae (L3) (red boxes) and molecules secreted from adult worms (blue boxes)

*Ancylostoma*-secreted proteins (ASPs) belong to a large family of cysteine-rich secretory proteins (CRISPS), called SCP/TAPS (SCP/Tpx-1/Ag5/PR-1/Sc7), which is recognized by the Pfam domain. The SCP/TAPS family includes mammalian sperm-coating glycoprotein, glioma-pathogenesis associated protein, lizard venom ryanodine-receptors blocker, hymenopteran venom antigen 5/3 family, plant pathogenesis-related proteins, and hyphae fungal proteins [12–14]. Multiple ASPs are secreted from both hookworm adults and infectious larvae (L3), as shown in Table 1 and Fig. 1. ASPs harbor a single or double SCP-like extracellular domain. Two ASPs, the double-domain *Ac*-ASP-1 and the single-domain *Ac*-ASP-2, were secreted from *A. caninum* L3 stimulated by host-specific signals in the early infection [15, 16]. *Ac*-ASP-1 was the most predominant protein released from the activated L3. The biological functions of larval ASPs are still unclear. However, the fast release of *Ac*-ASP-1 during host invasion and its partial similarity to insect venom allergens inferred its significance in hookworm pathobiology and immunobiology [15]. Additionally, *Ac*-*asp*-2 gene abundance among nematodes and its product release by host-specific signals show its importance in nematode development and physiology and probably in the infection process [16]. Several ASP family members were upregulated in serum-stimulated infectious larvae (ssL3) compared with the free-living one [17, 18]. Therefore, ASPs might be related to the early phase of hookworm transition to parasitism [18] and complementarily regulated upon stimulation by host serum [17].

**Table 1:** List of hookworm excretory/secretory products with an importance in host-parasite interactions

Molecule	GenBank	Size (aa)	Species	Stage	Location	Function	Reference
*Ac*-ASP-1	Q16937	424	*A. caninum*	L3	Unknown	Larval migration	[15]
*Ac*-ASP-2	AAC35986	218	*A. caninum*	L3	Unknown	Larval migration	[16]
*Ace*-ASP-1	AAN11402	425	*A. ceylanicum*	L3	Unknown	Larval migration	[19]
*Ace*-ASP-2	AAP41953	217	*A. ceylanicum*	L3	Unknown	Larval migration	[19]
*Na*-ASP-1	AAD13340	424	*N. americanus*	L3	Unknown	Larval migration	[15]
*Na*-ASP-2	AY288089	210	*N. americanus*	L3	Unknown	Larval migration and immunomodulation	[20, 22, 23]
*Ac*-ASP-3	AAO63575	200	*A. caninum*	Adult	Pharyngeal and esophageal glands	Unknown	[30]
*Ac*-ASP-4	AAO63576	508	*A. caninum*	Adult	Cuticle	Unknown	[30]
*Ac*-ASP-5	AAO63577	424	*A. caninum*	Adult	Intestinal microvilli	Unknown	[30]
*Ac*-ASP-6	AAO63578	451	*A. caninum*	Adult	Cephalic and excretory glands	Unknown	[30]
*Ac*-APc2	AAC47080	91	*A. caninum*	Adult	Esophagus	Factor VIIa/tissue factor complex inhibitor anticoagulant	[43, 50]
*Ac*-APc3	AAP57305	84	*A. caninum*	Adult	Esophagus	Factor VIIa/tissue factor complex inhibitor anticoagulant	[50]
*Ac*-APc4	AAP82926	99	*A. caninum*	Adult	Esophagus	Factor VIIa/tissue factor complex inhibitor anticoagulant	[50]
*Ac*-AP5	U30795	100	*A. caninum*	Adult	Cephalic glands	Factor Xa inhibitor anticoagulant	[43, 45]
*Ac*-AP6	AAC47081	98	*A. caninum*	Adult	Unknown	Factor Xa inhibitor anticoagulant	[43]
*Ac*-AP12	ADR77824	100	*A. caninum*	Adult	Esophagus	Factor Xa inhibitor anticoagulant	[51]
*Ac*-NAP10	ABP88128	90	*A. caninum*	Adult	Unknown	VIIa/tissue factor complex and Xia factor inhibitor anticoagulant	[52]
*Adu*-NAP4	ACD80355	104	*A. duodenale*	Adult	Unknown	Xa and Xia factors inhibitor anticoagulant	[53]
*Ace*-NAP1	AAK81733	102	*A. ceylanicum*	Adult	Unknown	VIIa/tissue factor complex and Xa factor inhibitor anticoagulant	[48]
*Ac*-HPI	AF399709	181	*A. caninum*	Adult	Cephalic glands	Platelet inhibitor	[47]
*Ace*-HPI	MK087839	200	*A. ceylanicum*	Adult	Esophagus; cephalic glands	Platelet inhibitor	[61]
*Ac*-APR-1	AAB06575	442	*A. caninum*	Adult and L3	Intestinal microvilli; esophagus; excretory/amphidial glands	Hb, serum, and connective tissue proteins digestion	[63, 64]
*Na*-APR-1	na	na	*N. americanus*	Adult and L3	Intestinal microvilli; esophagus; excretory/amphidial glands	Hb, serum, and connective tissue proteins digestion	[63, 66]
*Us*-APR-1	ACI02330	447	*U. stenocephala*	Adult	Unknown	Hb digestion	[67]
*Na*-APR-2	Q9N9H4	425	*N. americanus*	Adult	Gut lumen; amphidial and excretory glands	Hb and serum proteins digestion	[68]
*Ac*-CP-1	U18911	343	*A. caninum*	Adult	Esophageal, cephalic and excretory glands	Intestinal mucosal plug digestion	[70]
*Ac*-MTP-1	AAK62032	547	*A. caninum*	L3	Esophageal glands	Larval migration	[72, 73]
*Ace*-MTP-2	ABG49116	233	*A. ceylanicum*	Adult	Esophageal glands	Intestinal tissue degradation and immunomodulation	[74]
*Ace*-KI-1	AAD51334	84	*A. ceylanicum*	Adult	Subcuticle	Immunomodulation and host proteases inhibition	[78, 79]
*Ac*-KPI-1	AAN10061	759	*A. caninum*	Adult	Unknown	Unknown	[81]
*Adu*-TIL-1	GU951574	161	*A. duodenale*	Adult	Cuticle; esophagus; intestine	Neutrophil elastase and trypsin digestion	[82]
*Ac*-API-1	AAU29500	228	*A. caninum*	Adult	Pseudocoelomic fluid	Unknown	[87]
*Ac*-TMP-1	AF372652	140	*A. caninum*	Adult	Unknown	*Ac*-MTP-1 inhibitor and immunomodulator	[83]
*Ac*-TMP-2	ACB13195	244	*A. caninum*	Adult	Cephalic and esophageal glands	MMP inhibitors	[84]
*N*a-GST-1	FJ711440	206	*N. americanus*	Adult	Esophagus; muscles; hypodermis; gut	Heme and hematin detoxification	[90]
*N*a-GST-2	FJ711441	206	*N. americanus*	Adult	Buccal capsule; weakly in cuticle and gut	Heme and hematin detoxification	[90]
*N*a-GST-3	FJ711442	206	*N. americanus*	Adult	Buccal capsule; weakly in cuticle and gut	Heme and hematin detoxification	[90]
*Ac*-GST-1	AY605283	207	*A. caninum*	Adult	Hypodermis; muscle; weakly in intestine	Heme and hematin detoxification	[93]
*Ace*-GST	MN103336	155	*A. ceylanicum*	Adult	Cuticle; muscle; intestine	Heme and hematin detoxification	[94]
SOD	na	na	*N. americanus*	Adult	Unknown	Antioxidant	[104]
*Ace*-PRX-1	JX124321	196	*A. ceylanicum*	Adult	Unknown	Antioxidant and immunomodulator^a^	[89]
*Ac*-NIF	AAA27789	274	*A. caninum*	Adult	Unknown	Immunomodulation	[11]
*Ace*-MIF	ABO31935	119	*A. ceylanicum*	Adult and L3	Unknown	Immunomodulation	[120]
Calreticulin	AJ006790	403	*N. americanus*	Adult	Unknown	Complement binder and immunomodulator	[122, 123]
*Ace*-ES-1	AAL02424	109	*A. ceylanicum*	Adult	Unknown	Unknown	[124]
*Ace*-ES-2	AAS13463	102	*A. ceylanicum*	Adult	Unknown	Unknown	[125]
*Na*-CTL-2	AF388311	178	*N. americanus*	Adult	Unknown	Immunomodulation^a^	[131]
*Ace*-CTL-1	AF172652	184	*A. ceylanicum*	Male	Testis in male; embryo in female	Hookworm reproduction	[128]
*Na*-AChE	na	na	*N. americanus*	Adult	Amphidial and esophageal glands	Unknown	[133, 134]
*Ac*-FAR-1	AAM93667	181	*A. caninum*	Adult	Unknown	Fatty acid and retinol uptake	[143]
*Ac*-FAR-2	AAM97930	181	*A. caninum*	Adult	Unknown	Fatty acid and retinol uptake	[143]
*Ace*-FAR-1	EU449764	181	*A. ceylanicum*	Adult	Hypodermis; uterus; ovaries; testis	Fatty acid uptake for cuticle, egg and embryo development	[144]
*Na*-FAR-1	4UET	175	*N. americanus*	Adult	Intestine; male bursa	Nutrient uptake and male reproduction	[146]
*Ace*-NPA	na	na	*A. ceylanicum*	All stages	Pseudocoelom; cuticle; hypodermis; testis	Fatty acids uptake for cuticle integrity and male reproduction	[147]

^a^The immunomodulatory function of these molecules has been identified in mammals or other parasites and yet to be confirmed in hookworm

*Note*: This list includes only molecules whose cDNA or protein has been isolated, and excludes molecules exclusively identified as ESTs

*Abbreviation:* na, not available

*Ac*-ASP-1 and *Ac*-ASP-2 homologs were also isolated from the L3 stage of *A. ceylanicum* [19] and *N. americanus* [20]. The crystal structure of *Na*-ASP-2 revealed structural similarities to CC-chemokines, implying that *Na*-ASP-2 might imitate chemokines function during tissue invasion [21]. A further study recorded that *Na*-ASP-2 caused remarkable neutrophil recruitment [22]. Tribolet et al. [23] suggested that *Na*-ASP-2-induced neutrophil recruitment might help to suppress the host immunity against the parasite through two different but convergent ways because neutrophils are not only essential effector cells in acute infections but also can hinder B cell and T cell responses. Neutrophils can compete with antigen-presenting cells for antigen [24] and indirectly control T cell-dendritic cell interactions [25]. Moreover, *Na*-ASP-2 lowered the expression of genes involved in the B cell receptor signaling pathway by reacting with the cluster of differentiation CD79A on human B lymphocytes [23]. *Na*-ASP-2 antibodies in endemic areas were accompanied by lowering the risk of heavy necatoriasis. Recombinant ASP-2 raised antisera were potent *in vitro* inhibitors to L3 migration through tissue [26]. As a result of *Na*-ASP-2 protective and immunogenic characters, several studies have evaluated it as a human hookworm vaccine target [26–28]. However, this vaccine failed during the clinical trial due to its allergic potential [29].

In addition to ASPs secreted from hookworm larvae, adult *A. caninum* released four ASPs [30]. Meanwhile, the proteomic analysis of *A. caninum* ES products demonstrated that worms release over 30 distinct ASPs [31]. *Ac*-ASP-3, *Ac*-ASP-4, *Ac*-ASP-5 and *Ac*-ASP-6 showed distinct localization in pharyngeal and esophageal glands, cuticle, intestinal microvilli, and excretory and cephalic glands, respectively [30]. Three unique ASPs cDNAs (*Ace*-ASP-3-5) were cloned from *A. ceylanicum* by RACE-PCR technique. *Ace*-*asp*-3 and *Ace*-*asp*-5 transcription levels in serum-stimulated L3 larvae were lower than that in adult worms. Conversely, *Ace*-*asp*-4 was upregulated in ssL3 stages *versus* adult parasites [32]. Additional studies are required to express these genes as recombinant proteins and to test them as a possible vaccine and pharmaceutical target. Although the adult worm ASPs functions remain unknown, the release of multiple members and their abundance in adult hookworm ES products [33, 34] and intestinal transcriptomes [35, 36] imply their importance in host-parasite relationships. Furthermore, *Ancylostoma* species ASPs expression levels in males were higher than that in females [37], indicating that these proteins might play a role in male reproduction.

## Antithrombotics

As soon as the adult hookworm attaches to the intestinal mucosa, it lacerates mucosal blood vessels and sucks blood into its buccal capsule [38, 39]. To date, several structurally related hookworm antithrombotic compounds (Table 1, Fig. 1), including anticoagulants and hookworm platelet inhibitors, have been isolated from *A. caninum* and *A. ceylanicum* [40–48]. Other potentially related antithrombotic activities have also been identified in secretory products of adult *N. americanus* [49], but the cDNAs encoding these antithrombotic factors remain to be isolated and characterized. Together, the anticoagulants and platelet inhibitors act to maintain the adult worm’s blood-feeding ability. Thus, they might represent a potential vaccine target aiming to inhibit hookworm-related intestinal bleeding and iron deficiency anemia.

### Anticoagulants

Adult hookworms secrete a variety of anticoagulants, termed nematode anticoagulant peptides (NAPs), to allow the ingestion of blood liberated from lacerated capillaries. In *A. caninum*, three NAPs could inhibit coagulation factor VIIa/tissue factor complex, composed of *Ac*-APc2 [43], *Ac*-APc3 and *Ac*-APc4 [50]. Other NAPs from *A. caninum* inhibited factor Xa, including *Ac*-AP5 [41], *Ac*-AP6 [43] and *Ac*-AP12 [51], while *Ac*-NAP10 from *A. caninum* could inhibit both VIIa/tissue factor complex and Xia factor [52]. *Adu*-NAP4 from *A. duodenale* hindered both Xa and Xia factors [53], but *Ace*-NAP1 isolated from *A. ceylanicum* inhibited VIIa/tissue factor complex and fXa [48].

*Ancylostoma* anticoagulants, identified to date, have exhibited distinct mechanisms of action. Recombinant *Ac*-AP5 (r*Ac*-AP5) specifically inhibited the factor Xa through the interaction between the inhibitor’s P1 reactive site and the coagulation factor [41]. *Ac*-APc2 hindered the fVIIa/TF complex through a mechanism requiring the assembly of a quaternary complex (*Ac*-APc2-fVIIa-TF-fXa) for the maximal inhibitory activity [54, 55]. *Ac*-APc2 also inhibited the prothrombinase complex, probably *via* the interaction with coagulation factor Xa that does not involve the enzyme’s catalytic site. By contrast, *Ace*-AP1 differed from *Ac*-AP5 and *Ac*-APc2 in its inhibitory activity [46]. This anticoagulant used both active site and non-active site-mediated interactions to inhibit factor Xa [48]. Both *Ac*-APc3 and *Ac*-APc4 could inhibit fVIIa/TF only in the presence of fXa as an inhibitory scaffold, similar to that formerly described for *Ac*-APc2 [50].

Hookworm anticoagulants were immunolocalized to adult worms cephalic, or esophageal glands [45, 50, 51], and anticoagulant transcripts were identified in hookworm intestine cDNA libraries [35]. Thus, NAPs might facilitate blood-feeding by preventing thrombosis at the intestinal attachment site [56, 57] and maintaining the host blood liquid status while passing through the parasite gut [41]. Inhibitors of fVIIa/TF and fXa were localized to distinct structures within adult *A. caninum*, suggesting its complementary rather than redundant actions [50]. Hookworm anticoagulants represent a target for novel antithrombotic therapy. The anticoagulant NAPc2 from *A. caninum* decreased the development of deep vein thrombosis [58] and inhibited tumor growth and metastasis in mice [59].

### Hookworm platelet inhibitors

A powerful platelet inhibitor family, called hookworm platelet inhibitors (HPI), was isolated from soluble extracts of adult *A. caninum* [47]. HPI inhibited coagulation by hindering the platelet aggregation and their adherence to fibrinogen and collagen. This inhibitory action occurs *via* the blockage of the fibrinogen receptor integrin GPIIb/IIIa (*α*_IIb_*β*_3_) and the collagen receptor integrin GPIa/IIa (*α*_2_*β*_1_) [44]. Ac-HPI from *A. caninum* showed a significant similarity to other ASPs in the amino acid sequence [47] and crystal structure [60]. Despite its native structure, *Escherichia coli*-expressed recombinant *Ac*-HPI could not inhibit human platelet adhesion to fibrinogen or collagen. Consequently, *Ac*-HPI might need post-translational modification or exhibit a different biological role [60]. HPI native protein was detected in *A. caninum* extracts and ES products, and immunolocalized to the adult worm cephalic glands, indicating its release at the intestinal attachment site [47]. Recently, our group has cloned HPI from *A. ceylanicum* (*Ace*-HPI) with a 91% amino acid sequence homology to *Ac*-HPI. *Ace*-*hpi* transcripts were most abundant in adults, followed by ssL3s and L3 stages, with a significant difference. Unlike *Ac*-HPI, *E. colli*-expressed recombinant *Ace*-HPI protein inhibited the platelet aggregation [61], supporting the role of *Ace*-HPI in hookworm blood-feeding.

## Proteases

A large family of hookworm proteases was highly represented in ES products of *A. caninum* [31] and *N. americanus* [34]. These proteases belonged to the three nematode proteases classes (aspartic, cysteine and metalloproteases), presented in Table 1 and Fig. 1. Many hookworm proteases have been contributed to the digestion of free hemoglobin (Hb), thus called hemoglobinases, through a multi-enzyme-synergistic cascade of proteolysis [62]. These hemoglobinases are mostly attached to the adult worm gut and not secreted in ES products. Hence, we do not discuss them in this review.

### Aspartic proteases

Cathepsin D-like aspartic proteases from *A. caninum* (*Ac*-APR-1) and *N. americanus* (*Na*-APR-1) cleaved intact Hb at numerous distinct sites [63, 64]. Several studies have shown that these proteases act functions in the hookworm biology other than blood-feeding. *Necator americanus* larvae secretion had aspartic protease activity that digested skin macromolecules (fibronectin, collagen, elastin and laminin). Hindering this activity with pepstatin A inhibited larval migration through the skin [65]. *Ac*-APR-1 and *Na*-APR-1 mRNAs were expressed in both L3 and adult stages of *A. caninum* and *N. americanus* [63]. Later, it had been demonstrated that both proteases can also digest skin macromolecules and serum proteins. Some substrates from permissive definitive hosts were cleaved more easily than those from non-permissive hosts [66]. Williamson et al. [66] linked this difference in substrate preferences to the conformational difference in the S3 pocket residues. *Ac*-APR-1 and *Na*-APR-1 raised antisera partially inhibited hookworm L3 migration *via* hamsters’ skin, indicating these enzymesʼ roles in the invasion of host connective tissue [66]. *Us*-APR-1 was cloned from hookworm *Uncinaria stenocephala* using the RACE-PCR method, yielding a 52-kDa protein homologous to APRs from related hookworms. Western blotting using *Us*-APR-1 raised antisera identified *Us*-APR-1 protein in the adult *U. stenocephala* extract and its homologous protease in *A. ceylanicum* L3 and adult extract and ES products [67]. *Us*-APR-1 cross-reactivity to its homolog in *A. ceylanicum* might help to develop a single recombinant-APR vaccine to cross-protect against several canine hookworms [67]. The presence of *Us*-APR-1 in *U. stenocephala* ES products has not been tested, but it contains a signal peptide, suggesting that it is a secretory protein. Although genes encoding *A. ceylanicum* APR-1 predominated in *A. ceylanicum* intestinal transcriptome [36], more studies are required to clone, express and characterize this enzyme.

A distinct aspartic protease isolated from *N. americanus* (*Na*-APR-2) was more related to nematode-specific aspartic proteases (nemepsins) family than to cathepsin D. *Na*-APR-2 was detected only in the gut lumen and amphidial and excretory glands of adult worms [68], indicating its secretory nature. The ability of recombinant *Na*-APR-2 to digest Hb and serum proteins from humans was nearly twice its ability to digest that from the nonpermissive host (dog). *Na*-APR-2 raised antiserum inhibited 50% of *N. americanus* L3 migration in the skin [68], which stimulates the further investigation of this enzyme as a vaccine candidate.

### Cysteine proteases

Hookworm secretions exhibited cysteine proteinase activity that degraded the synthetic cathepsin L-specific substrate (Z-Phe-Arg-AMC) but not the cathepsin B and H substrates (Z-Arg-Arg-AMC and Z-Arg-AMC) [69]. Later, a cathepsin B cysteine protease (*Ac*-CP-1) was cloned and sequenced from adult *A. caninum* [70], but no studies identified cathepsin L cysteine protease genes. *Ac*-CP-1 crystal structure differed from that of human cathepsin B in the substrate binding and specificity; *Ac*-CP-1 more favorably cleaved Phe-Arg than Arg-Arg. These results explain why the hookworm CPs, although structurally similar to cathepsin B, show a cathepsin L-like activity [71]. *Ac*-CP-1 was localized in esophageal, cephalic and excretory glands [70], identified in ES products [31], and showed protease activity [70]. Thus, *Ac*-CP-1 might promote the digestion of intestinal mucosal plug engulfed by the parasite at the attachment site.

### Metalloproteases

Metalloproteases are among the highly represented proteins in ES products of adult hookworms [31]. Hookworm metalloproteases secreted as ES products mainly belong to astacin-like zinc-metalloprotease (MTP) class. The activated *Ancylostoma caninum* L3 secretes *Ac*-MTP-1 that aids in the larval tissue migration by hydrolyzing fibronectin, gelatin, laminin and collagen [72, 73]. *Ac*-MTP-1 was immunolocalized to glands in the esophagus and the channels connecting the esophagus to the cuticle, and its specific antisera inhibited larval migration [72]. These findings support the role of *Ac*-MTP-1 in the larval migration and the infection process. Additionally, adult hookworm secretes astacin-like zinc metalloproteases, including *Ac*-MTP-2 from *A. caninum* and *Ace*-MTP-2 from *A. ceylanicum. Ac*-MTP-2 showed a proteolytic activity and was localized to the esophageal glands. Hence, it is suggested to degrade the intestinal plug engulfed in the adult worm buccal capsule [74]. *In vitro*, *Ace*-MTP-2 stimulated macrophages to release TNF*α* and IFN*γ* [75], indicating its function in host-parasite interactions.

## Protease inhibitors

Adult hookworms produce multiple protease inhibitors, which were highly represented in the transcriptome of adult hookworms such as *N. americanus* [76]. Hookworm protease inhibitors can be classified into Kunitz-type protease inhibitors, trypsin inhibitor-like protein, tissue inhibitor of metalloprotease, and aspartyl protease inhibitors (Table 1, Fig. 1). Some of these protease inhibitors are involved in disease pathogenesis.

### Kunitz-type protease inhibitors

Kunitz-type protease inhibitors (KIs) are widely distributed among eukaryotes. These protease inhibitors carry single or multiple cysteine-rich Kunitz domains and display inhibitory activity against one or more serine proteases, including pancreatic trypsin [77]. Adult hookworm ES products showed a KI-similar inhibitory activity, inhibiting pancreatic elastase, trypsin and chymotrypsin [78]. This finding suggests that protease inhibitors are secreted by adult worms at the site of attachment probably to aid in the parasite survival and the hookworm-related anemia pathogenesis by hindering host intestinal proteases [78]. Later, a 7.9-kDa *Ace*-KI-1, with a single Kunitz domain, was isolated from *A. ceylanicum* and showed a potent-binding inhibitory action on neutrophil elastase, trypsin, chymotrypsin and pancreatic elastase. This unique, broad-spectrum activity indicates that *Ace*-KI-1 contributes to both immune modulation and protection against host intestinal proteases [78]. *Ace*-KI-1 was immunolocalized to the adult hookworm subcuticle, supporting its *in vivo* role in inhibiting intestinal proteases on worm surfaces [79]. A large multiple domain KI (84.9 kDa) was cloned and expressed from adult *A. caninum* (*Ac*-KPI-1). Both *Ace*-KI-1 and *Ac*-KPI-1 had a hydrophobic signal peptide and lacked a transmembrane domain in their deduced amino acid sequences, suggesting that these molecules are secreted [80]. *Ac*-KPI-1 contained 12 Kunitz domains with a high similarity to extracellular matrix proteins contributing to insect cellular remodeling and morphogenesis [81]. However, the exact role of this protein is yet to be identified.

Along with *Ace*-KI-1 and *Ac*-KPI-1, cDNA encoding two KPI types, single- (1D) and multiple-domain (12D) KPI were isolated from *Ancylostoma* species. These KPI comprised *Ab*-KPI (1D) and *Ab*-KPI (12D) from *A. braziliense*, *Ace*-KPI (1D) from *A. ceylanicum* and *Ac*-KPI (1D) from *A. caninum*. The transcription of both types of KPI was higher in males than in females [37], suggesting the possible role of these molecules in male reproduction. In addition to serine-protease inhibitors’ role in hookworm protection from the host intestinal proteolysis, they have been linked to the hookworm-related malnourishment and growth delay. This hypothesis was supported by immunization studies on *Ace*-KI-1 [79].

### Trypsin inhibitor-like protein

A distinct member of serine protease inhibitors (*Adu*-TIL-1) was cloned from *A. duodenale*. *Adu*-TIL-1 contained two trypsin inhibitor-like (TIL) domains similar to the *Ascaris* family of serine protease inhibitors. This protease inhibitor was localized to the cuticular surface, esophagus, and intestine of the adult worms [82]. Although *Adu*-TIL-1 detection in ES products has not been investigated, the detection of a signal peptide in its sequence supports its extracellular secretion by the adult worm. The two TIL domains of *Adu*-TIL-1 showed varying inhibitory activities against human neutrophil elastase and pancreatic trypsin, suggesting its role in *A. duodenale* survival [82]. Further studies to characterize this molecule in other hookworms are necessary.

### Tissue inhibitor of metalloprotease

Mammals secrete many matrix metalloproteases (MMPs) from immune cells and gut epithelium at the inflammation site. These metalloproteases help in remodeling extracellular matrix and are controlled by tissue inhibitors of metalloproteases (TIMPs). TIMPs are the major proteins secreted by adult hookworms, accounting for 6% of the total ES products [83]. Among them, *Ac*-TMP-1 (16 kDa) and *Ac*-TMP-2 (27.7 kDa) were isolated from adult *A. caninum* ES products [83, 84]. Further evidence suggested that hookworm-secreted TIMPs are involved in the immune-modulation of host immune system, which has been used to treat allergic or auto-immune diseases as major components of helminth therapy. Therefore, *Ac*-TMP-2 was re-named as *Ac*-AIP-2 (anti-inflammatory protein) for the treatment of asthma [85]. No MMP-inhibitory action has been recorded for *Ac*-TMP-1, whereas it hindered an endogenous metalloprotease, *Ac*-MTP-2 [74]. *Ac*-TMP-1 stimulated dendritic cells to release inflammatory cytokines and differentiate into suppressor T cells (CD4+ and CD8+) [86], indicating its immunosuppressive role. *Ac*-TMP-2 was localized to the adult parasite cephalic and esophageal glands [84], enhancing the theory about *Ac*-TMPs release to function at the attachment site [7]. Recombinant *Ac*-TMP-2 was highly immunoreactive and inhibited several human MMPs, such as MMP-2, MMP-7 and MMP-13 [84], but its function in host immune regulation remains unclear.

### Aspartyl protease inhibitors

Nematodes aspartyl protease inhibitors are highly immunoreactive and have been proposed as potential vaccine targets. A member of the nematode aspartyl protease inhibitors (Aspins) family, named aspartyl protease inhibitor (API-1) was cloned from *A. caninum* and *A. ceylanicum* L3. *Ac*-*api*-1 mRNA was detected in all life-cycle stages, but the protein was only translated in adult *A. caninum* ES products. *Ac*-API-1 was mainly localized in the adult hookworm pseudocoelomic fluid. Soluble, yeast-expressed *Ac*-API-1 could not inhibit a hookworm aspartyl protease or pepsin but inhibited nearly 30% of the proteolytic activity in adult ES products. These data suggest that *Ac*-API-1 hinders an unknown, putative aspartyl protease from adult hookworms or may be secreted as an enzyme-inhibitor complex [87]. Thus, the substrate of this molecule needs to be determined.

## Antioxidants

The degradation of hemoglobin (Hb) releases iron-carrying heme and hematin, that can be lethal to hookworm through the production of reactive oxygen species (ROS) [88]. ROS are also released from host immune effector cells in their response to hookworm infection [89]. Therefore, hookworm secretes multiple antioxidant compounds, which might protect the worm exposed cuticular surfaces through neutralization of ROS generated from Hb digestion and host immune cells.

### Glutathione S-transferases

Glutathione S-transferases (GSTs) have been suggested to detoxify the iron-carrying heme and hematin by creating homodimers to chelate these compounds. GSTs (*Na*-GST-1, *Na*-GST-2 and *Na*-GST-3), with a high heme-binding affinity, were isolated from *N. americanus* extracts and ES products [90]. Crystallography of *N. americanus* GSTs showed that these molecules can homodimerize in solution to produce large heme-binding cavities [91]. Goud et al. [92] expressed *Na*-GST-1 in the yeast *Pichia pastoris* and purified it by three chromatography phases, producing a 23.7-kDa protein with a 51% yield and a 98% purity. Furthermore, a 24 kDa *Ac*-GST-1 was cloned from adult *A. caninum* [93], and its homologous enzyme has been isolated from *A. ceylanicum* [94]. *Ac-gst*-1 mRNA was detectable in all developmental stages, but its protein was only detectable in adult worm extracts and ES products [93]. These results indicate that this mRNA might be transcribed but not expressed or expressed at undetectable levels in non-blood feeding phases. *Ac*-GST-1 was shown to localize to the hookworm hypodermis and muscles and mildly to the intestine. The recombinant enzyme showed a high-affinity hematin binding site [93]. GSTs proposed function in heme detoxification triggered several studies to evaluate them as vaccine antigens [28, 90, 93, 95].

### Superoxide dismutases

Parasites also produce antioxidant enzymes called superoxide dismutases (SODs), which catalyze the conversion of superoxide anions into hydrogen peroxide and molecular oxygen. The toxic hydrogen peroxide can be removed by several enzymes, including peroxidase, glutathione peroxidase and catalase, which were detected in adult *A. ceylanicum* extracts [96]. Complementary DNAs encoding SODs have been cloned from multiple parasitic nematodes, such as *Onchocerca volvulus* [97], *Haemonchus contortus* [98], *Brugia* [99] and *Toxocara canis* [100]. Activated leukocytes release ROS, including superoxide anion, as a part of their antiparasitic activity. Thus, it has been proposed that SOD is expressed by parasitic helminths as an enzymatic defense against ROS immune-effector mechanisms [101, 102]. Nitric oxide interacts with superoxide anions to produce the hydroxyl radical. Therefore, parasite SOD might act as a defensive link between host nitric oxide and ROS-dependent attack because it might reduce host nitric oxide-dependent hydroxyl radical production by mopping up host-derived superoxide anions [103]. Cu/Zn SOD activity was detected in *N. americanus* ES products [104] and adult *A. ceylanicum* extract [96]. However, further research is necessary to clone the cDNAs encoding these proteins from hookworms and to elucidate their biological functions.

### Peroxiredoxins

Peroxiredoxins (PRXs), also called peroxidoxin or thiol-specific antioxidants, are antioxidants conserved in prokaryotes and eukaryotes. Hookworms secrete peroxiredoxins, a family of peroxidases important for protection against ROS. Parasite PRXs can also act as signaling molecules and chaperones [90]. A peroxiredoxin (*Ace*-PRX-1) was cloned from *A. ceylanicum* (Table 1, Fig. 1). *Ace*-*prx*-1 transcript was more abundant in adults than in eggs and larvae, and its protein was only detected in adult worm extracts and ES products [90]. Thus, *Ace*-*prx*-1 mRNA might be translated only in the blood-feeding stages, the only exposed stages to oxidation by heme. Parasite PRXs can detoxify extremely reactive oxygen species, such as hydroxyl radicals and hydrogen peroxide, and reactive nitrogen species [105], suggesting that hookworm PRXs defensive role is complementary to SOD protective role by detoxifying SOD produced hydrogen peroxide. Although PRXs secreted from other helminths (*Schistosoma mansoni* and *Fasciola hepatica*) were suggested to stimulate macrophage activation and Th2 helper cell differentiation [106], the immunomodulatory role of secreted *Ace*-PRX-1 remains to be explored.

## Immunomodulators

Adult hookworms secrete many molecules with a potential immunomodulatory function, such as neutrophil inhibitory factor, macrophage migration inhibitory factor and calreticulin (Table 1, Fig. 1). These molecules might inhibit the inflammatory reaction, promote effector cells apoptosis and skew the immune response phenotype; as a result, they can help the parasite survival inside the host [8]. Recently, hookworm ES products have been evaluated as a therapeutic target. These products contain immunoregulatory properties, which can suppress inflammatory diseases such as inflammatory bowel diseases, colitis, asthma and other allergic diseases (reviewed by Navarro et al. [10]).

### Neutrophil inhibitory factor

Neutrophil inhibitory factor (NIF) is a novel, 41 kDa cysteine-rich glycoprotein, with no significant sequence similarity to any previously identified protein, was isolated from adult *A. caninum* [11]. It might be used by the hookworm to minimize the local inflammation so that the parasite is protected from the host immune response. Hookworm NIF is a specific *β*_2_-integrin CR3 antagonist, which bound neutrophils through the CD1lb A-domain, preventing them from recognizing CR3 ligands and mediating phagocytosis [107]. Also, *Ac*-NIF could inhibit CD11b/CD18-dependent neutrophil function by preventing the activated neutrophils from adhering to vascular endothelium and releasing H_2_O_2_ [11]. Further studies showed that recombinant *Ac*-NIF (r*Ac*-NIF) could block the adherence of neutrophils to fibrinogen by binding the neutrophil I domain of CD11b/CD18 [108]. Additionally, NIF hindered the aggregation of phorbol ester-activated JY lymphoblastoid cells, only expressed in the active CD11a. Thus, NIF antiadhesive action might be caused by inhibition of both CD11a and CD11b *β*_2_ integrins on neutrophils [109]. Although divergent homologs of NIF were identified as expressed sequence tags (ESTs) from adult *N. americanus* [110], their roles have not been explored yet.

*Ac*-NIF has been studied as a possible therapeutic agent. The *Ac*-NIF expression caused *β*-Integrin blockage, which prevented neutrophil activation, migration and sequestration, and subsequently prevented tissue inflammation and lung injury in mice [111]. A combination of r*Ac*-NIF and anti-CD11a/CD18 monoclonal antibody significantly reduced neutrophil accumulation and moderately decreased dermal edema in rats with a reverse passive Arthus reaction [112]. In a rodent focal cerebral ischemia model, r*Ac*-NIF administration for 48 h had a significant neuroprotective action [113]. This action was caused by lowering neutrophil number within the tissue, suggesting r*Ac*-NIF potential therapeutic properties in stroke [113]. Additionally, *Ac*-NIF selectively inhibited the trans-endothelial migration of eosinophils *in vitro* and air passages eosinophilia in OVA-induced lung allergic reaction [114].

### Macrophage migration inhibitory factor

Macrophage migration inhibitory factor (MIF) is a pro-inflammatory cytokine originally detected as an activated T cell product and then revealed to exert various biological functions [115]. Mammalian MIF inhibits macrophages’ random migration and can counteract anti-inflammatory and immunosuppressive effects of glucocorticoids [116]. MIF stimulates cytokines release from macrophages, T cell proliferation, and the secretion of nitric oxide, matrix metalloproteases, cyclooxygenase 2 and prostaglandin E2 [117–119]. An ortholog of the human MIF was isolated from *A. ceylanicum* (*Ace*-MIF). *Ace*-MIF could antagonize the mammalian MIF for attaching to its cell surface receptor CD74. Consequently, *Ace*-MIF is assumed to modulate the host immune responses against skin-penetrating larvae and adult worm attached to the small intestine. Immunoblotting detected the native *Ace*-MIF in L3 and adult worm extracts and ES products, the parasitic stages that must challenge host immunity [120]. These data provide a probable tool for reducing hookworm survival and disease pathogenesis through selective inhibition of the hookworm cytokine. However, further research is required to isolate and characterize the homologs of this protein in other hookworm species.

### Calreticulin

Calreticulin is a highly conserved and multifunctional calcium-binding protein, which is present in all cells of higher creatures, except erythrocytes. Numerous calreticulin-associated functions in parasites were reviewed by Ferreira et al. [121]. These functions include calcium storage, chaperoning, integrin-mediated signaling and cell adhesion, modulating gene expression, as well as inhibiting T cells and natural killer cells perforin pore formation, tumor growth, angiogenesis, and C1q-dependent complement activation [121]. Hookworm calreticulin was originally identified as a key allergen reacting with IgE from *N. americanus*-infected patients [122]. Later, calreticulin was cloned, expressed, and purified from adult *N. americanus* extracts. Although calreticulin transcripts were detected in L3, L4 and adult stages, the protein was only detected in adult worm extracts and ES products. Recombinant calreticulin was a specific binding-inhibitor for the hemolytic capacity of human complement C1q [123]. Therefore, calreticulin might play a role in inhibiting the host immune reaction through blocking complement activation. However, further studies are required to isolate and characterize this molecule from *Ancylostoma* species.

## Other ES products

### Excretory/secretory proteins

A unique 12.9 kDa major secretory protein, called *A. ceylanicum* excretory/secretory protein 1 (*Ace*-ES-1), was purified and cloned from adult *A. ceylanicum* (Table 1). *Ace*-ES-1 was more abundant than other proteins in *A. ceylanicum* ES products, indicating its possible key role in disease pathogenesis. Although *Ace*-ES-1 was strongly immunogenic and reacted to *A. ceylanicum*-infected hamster serum [124], its biological role is still unclear. Thereafter, an 11.7 kDa protein (*Ace*-ES-2) was cloned from adult *A. ceylanicum* ES products (Table 1). This protein showed a netrin-like fold similarity to that of the TIMP family [125, 126]. Adult worms secreted *Ace*-ES-2 at the intestinal attachment site soon after infection [125] and before parasites feed on blood [127]. Although *Ace*-ES-2 had no significant inhibitory effect on MMPs or complement-mediated cell lysis, the highly acidic surface on *Ace*-ES-2 implies its potential role as a cytokine decoy receptor [126]. *Ace*-ES-2 was highly immunogenic in infected animals and induced protection when administered as an oral vaccine [125], but whether this molecule has an immunomodulatory function needs to be analyzed.

### C-type lectins

Lectins represent a family of related proteins that regulate crucial cell functions *via* attaching to carbohydrates. One member of this family is C-type lectins (C-TLs), which are recognized by their calcium requirements for the optimal biological activity [128]. This sugar-binding protein family (C-TLs) serves as glycoprotein ligand receptors. C-TLs are located in serum and on endothelial and immune cells (B and T cells) to share in orchestrating the immune system [129]. Proteomic analysis of *A. caninum* ES products identified lectins, including three C-type lectins, as one of the highly represented proteins [31]. C-TLs transcripts, with a sequence homology to host immune system lectins (CD23 and P-selectin), were identified in adult *N. americanus* [110, 130]. Additionally, analysis of *N. americanus* and *A. caninum* intestinal transcriptomes reported C-type lectins as abundant transcripts [35]. A cDNA encoding a secreted C-TL (*Na*-CTL-2) was cloned from *N. americanus* and expressed only in adult worms [131].

C-TLs might inhibit the local inflammatory reaction to hookworm feeding in the intestine by antagonizing host lectins for attaching to inflammatory ligands [8]. Another hypothesis is that C-TLs in hookworms and other hematophagous parasites might have an anticoagulant activity similar to C-TLs in snake venom [8]. Moreover, a male-sex-specific C-type lectin was cloned from *A. ceylanicum* (*Ace*-CTL-1) and could bind N-acetyl-d-glucosamine (a constituent of eukaryotic egg cell membranes) *in vitro*. *Ace*-CTL-1 was detected in male extracts and sperms and immunolocalized to the testis in males and growing embryos in females [128], suggesting its role in reproduction. Recently, the blockage of CTL receptors on human dendritic cells (DC) has interfered with *N. americanus* larval exsheathment and DC sequestration around the sheath. These data showed DC-CTL role in the L3 exsheathment and evasion from DCs immune recognition [132]; however, the effect of hookworm-derived C-type lectins on host DC and larval exsheathment is yet to be determined.

### Acetylcholinesterase

All nematodes, either parasitic or free-living, carry cholinergic motor neurons, so they may use acetylcholinesterase (AChE) to lysis acetylcholine [8]. In addition to neuronal AChE, many parasitic nematodes, including hookworms, secrete AChE into the *in vitro* culture medium [133]. Immunohistochemistry has confirmed that the enzyme is originated from cephalic and esophageal glands of adult hookworms [133, 134]. AChE purified from adult *N. americanus* ES products showed a specific activity on acetylthiocholine iodide [133] and was homologous to *Caenorhabditis elegans* AChE class B [135]. Nevertheless, hookworm AChE should be carefully tested against a wider spectrum of substrates compared with free-living nematode AChE [136]. Western blotting demonstrated a significant difference in reaction to AChE-immunized rabbit serum between ES products of *N. americanus* males and females [133]. This sex difference suggests that AChE might help in, or be modified by, genital tract secretions [136], but this role has not been confirmed yet.

Although AChE release from parasitic nematodes is well accepted, its biological functions are argumentative [133]. Several functions have been anticipated for nematode nonneuronal AChE. This enzyme might play a role in host gut peristalsis inhibition, enteric secretory mechanisms control [137], immunomodulation, cell development, intestinal wound healing, and choline production for biosynthesis [138]. AChE immunogenicity was first confirmed in a volunteer inoculated with four doses of *N. americanus* L3 over 27 months [139]. AChE-specific antibodies were identified 12 weeks following the second dose, boosted by following infections and dropped after treatment [139], which excluded AChE as a vaccine candidate [136]. Furthermore, AChE activity was identified in *A. caninum* and *A. tubaeforme* [140, 141] but at a concentration significantly lower than those in *N. americanus* [142]. The three hookworm species have a similar impact on host gut peristalsis, indicating that AChE might not inhibit gut motility but might act a yet unidentified function [136]. AChE genes were cloned from the rodent nematode *Nippostrongylus brasiliensis* [137], while there are no available data about the cloning of AChE from hookworms.

### Fatty-acid and retinol-binding proteins

Nematodes, including hookworms, produce two distinct classes of fatty acid- and retinol-binding proteins (FAR, fatty acid- and retinol-binding; and NPA, nematode polyprotein antigen/allergen), as shown in Table 1, Fig. 1. These proteins might help the uptake, transfer, and metabolism of sterols, fatty acids and retinol in the parasites. FAR mRNAs were highly presented in hookworm intestinal transcriptomes [35]. *Ac*-FAR-1 and *Ac*-FAR-2 cDNAs were first cloned from a cDNA expression library using *A. caninum* ES products-immunized rabbit serum [143]. Then, *Ace*-FAR-1 and *Na*-FAR-1 cDNAs were isolated from adult *A. ceylanicum* [144] and *N. americanus*, respectively [145]. *Ac*-FAR-1 protein was only detectable in adult worm extracts and ES products [143]. *Ace*-*far*-1 mRNA was most abundant in eggs, while the protein was expressed in both sexes, only detectable in female ES products and localized in the hypodermis, ovaries, uterus, and testis. These data show that *Ace*-FAR-1 might help fatty acid uptake for cuticle integrity, egg generation, and embryo development [144]. On the contrary, *Na*-FAR-1 was localized in the male copulatory bursa and the adult worm intestine, indicating their roles in male reproduction and nutrient uptake [146]. FAR might promote the parasite infectivity and lower the intestinal mucosal IgA levels through decreasing the retinol amount required for repairing the intestinal injuries and upregulating Th2 cytokine response, respectively [143]. To identify the exact function of the hookworm FARs, further studies are required to determine what FARs bind.

A partial cDNA was cloned from the four subunits of a putative *A. ceylanicum* NPA (*Ace*-NPA). *Ace*-NPA protein was detectable in extracts from all life stages and ES products from both male and female nematodes. Unlike r*Ace*-FAR-1, *Ace*-NPA protein extracts from L3 and adult extract were more immunoreactive than that from eggs [147]. *Ace*-NPA were localized to the cuticle, hypodermis and the male testis but not in the female, proposing its role in obtaining fatty acids required for the cuticle integrity and male reproduction. A single recombinant *Ace*-NPA subunit (r*Ace*-NPAb) bound fatty acids with C12-C22 chain [147] but with affinity different from that of r*Ace*-FAR-1 [144]. This difference in *Ace*-NPA expression, localization, and binding affinity from that of *Ace*-FAR-1 indicates a distinct function for both proteins in hookworm biology [147]. However, the functional difference between *Ace*-NPA and *Ace*-FAR has not been fully understood yet.

## Targets for vaccine and therapy

Strategies are frequently implemented to control hookworm infection in humans and animals due to the high morbidity caused by this infection [148]. In endemic areas, hookworm control depends on the regular treatment with benzimidazole anthelmintic drugs [149]. Unfortunately, anthelmintic drugs currently fail to control hookworm infection due to rapid re-infection and emerging benzimidazole resistance [32]. These facts have focused the attention on establishing an efficient vaccine using several hookworm ES products (Table 2).

**Table 2:** Summary of vaccine trials using hookworm excretory/secretory products

Vaccine antigen	Size (kDa)	Vaccine formulation	Delivery route	Effect	Target species	Development stage	Reference
*Ac*-ASP-1	42.0	Alum-precipitated RP	IP	Larval migration inhibition	*A. caninum*	Animal trial	[150]
*Ac*-ASP-2	24.2	RP/Glaxo SmithKline adjuvant	IM	Larval migration inhibition	*A. caninum*	Animal trial	[26]
*Ace*-ASP-2	24.0	RP/Quil A	IM	Larval migration inhibition	*A. ceylanicum*	Animal trial	[19]
*Na*-ASP-2	21.3	RP/Alhydrogel	IM	Larval migration inhibition	*N. americanus*	Phase 1 clinical trial	[28]
*Ac*-MTP-1	61.0	RP/Glaxo SmithKline adjuvant	IM	Larval migration inhibition	*A. caninum*	Animal trial	[155]
*Ac*-APR-1	~ 50.0	RP/Glaxo SmithKline adjuvant	IM	Reduction of anemia, worm burden, and egg count	*A. caninum*	Animal trial	[156]
		RP/Alhydrogel	SC	Worm burden reduction	*N. americanus*	Animal trial	[28]
Mutant *Na*-APR-1	na	Mutant protein /Alhydrogel	IM	Reduction of anemia, weight loss, and egg counts	*A. caninum*	Animal trial	[158]
*Ac*-GST-1	30.0	RP/Glaxo SmithKline adjuvant	IM	Worm burden and egg count reduction	*A. caninum*	Animal trial	[93]
		RP/Alhydrogel	SC	Worm burden and egg count reduction	*N. americanus*	Animal trial	[28]
*Na*-GST-1	24.0	RP/Alhydrogel	IM	Worm burden reduction	*N. americanus*	Phase 1 clinical trial	[90, 95]
*Ace*-KI-1	7.9	RP/Freund’s adjuvant	SC	Malnutrition and growth delay prevention	*A. ceylanicum*	Animal trial	[79]
Calreticulin	56.0	RP in phosphate buffer saline	IP	Worm burden reduction in lungs	*N. americanus*	Animal trial	[161]
*Ace*-ES-2	11.6	RP in 1.5 M NaCl	PO	Decreasing anemia and improving the recovery rate	*A. ceylanicum*	Animal trial	[125]
*Ace*-FAR-1	20.0	RP/cholera toxin	PO	Worm burden reduction	*A. ceylanicum*	Animal trial	[144]

*Abbreviations:* na, not applicable; RP, recombinant protein; IP, intraperitoneal injection; IM, intramuscular injection; SC, subcutaneous injection; PO, *per os* (orally)

The early developed irradiated hookworm larval vaccine was removed from markets due to non-sterile immunity and difficult wide-scale production and distribution [8]. Hence, hookworm larval secretions, including the ASP family, constituted the next focus of interest for developing a hookworm vaccine. Recombinant *Ac*-ASP-1 (r*Ac*-ASP-1) vaccine reduced hookworm burdens in mice lungs and muscles, suggesting that this vaccine produced antibodies that inhibit larval migration [150]. This study was the first to shed light on this family of proteins as vaccine targets. Following that, cross-species protection against *A. caninum* L3 challenge was induced using rASP-1 from the human hookworms, *A. duodenale* and *N. americanus*. This cross-protection was proportionally correlated to the sequence homology percentage between the vaccine ASP and the challenge larvae *Ac*-ASP-1 [151]. Conversely, *Ac*-ASP-2, with a 55% amino acid sequence homology to *Ac*-ASP-1, did not induce protection [151]. Hamsters vaccinated with recombinant *Ace*-ASP-2 (*Ay*-ASP-2) from *A. ceylanicum* showed significant decreases in worm burdens and the length of recovered worms was decreased [19]. Some studies have also investigated ASP from *N. americanus* (*Na*-ASP-2) as a lead vaccine candidate. In Sprague Dawley rat, vaccination with *Na*-ASP-2 formulated with aluminum hydroxide adjuvant (Alhydrogel) resulted in a strong and durable antibody response [152]. Moreover, the air pouch of vaccinated rats demonstrated increased levels of antigen-presenting cells and cytokines (IL-4, IL-10, IFN-γ and IL-5). Hence, this vaccine might neutralize the parasite at the inoculation site, preventing its migration into the host tissue [27]. In golden hamsters, *Na*-ASP-2/Alhydrogel vaccine lowered worm burdens [28]. Similarly, dog vaccination with *rNa*-ASP-2/Glaxo SmithKline adjuvant significantly decreased worm burdens and fecal egg counts through the release of antibodies, which inhibited larval migration [26]. It has been found that the anti-*Na*-ASP-2 IgE level in an endemic population was correlated with low hookworm burdens, indicating the protective association between increasing anti-ASP-2 IgE levels and the risk of heavy hookworm infection [26]. A phase 1a vaccine trial in hookworm-non-exposed adults from the USA showed that *Na*-ASP-2 vaccine was well-tolerated and induced a prolonged immune response [153]. By contrast, a phase 1b trial in hookworm-exposed individuals in Brazil was stopped due to the generalized urticaria developed in several volunteers harboring pre-formed *Na*-ASP-2-specific IgE [29]. Despite its safety alarms, *Na*-ASP-2 remains as an interesting vaccine candidate, especially if it is genetically modified to decrease its allergenicity or utilized as a pediatric vaccine before anti-hookworm IgE development [154].

Along with ASP-1 and ASP-2, the effect of immunization with another larval antigen *Ac*-MTP-1 was studied. Dogs vaccinated with r*Ac*-MTP-1 developed pre-challenge *Ac*-MTP-1-specific IgG2 and IgE antibody response. After the L3 challenge, hookworm burdens and egg counts were inversely correlated to anti-*Ac*-MTP-1 IgG2 antibody titers, with a statistical significance, proposing that this protein provides a hopeful vaccine candidate [155].

Recently, recombinant vaccines targeting adult hookworm proteases have shown a high efficacy. A recombinant *A. caninum* aspartic protease (r*Ac*-APR-1) induced significant protection in dogs, with reducing anemia, hookworm burdens, and egg counts [156]. Interestingly, r*Ac*-APR-1 vaccine in dogs decreased adult hookworm number obtained from the small intestine and increased the worm burden from the large intestine, an unusual hookworm habitat. This change in the habitat preference proposes that *Ac*-APR-1-induced antibodies hindered the blood-feeding in the small intestine and thus forced worms to migrate into a more favorable habitat [157]. Worm burdens significantly dropped in *Ac*-APR-vaccinated hamsters challenged with a different hookworm species (*N. americanus*) L3 [28], suggesting that *Ac*-APR-1 might cross-protect against its orthologs from other hookworm species. The enzymatically inactive mutant *Na*-APR-1 partly protected dogs against *A. caninum* challenge, especially from blood loss, with significantly declined weight loss and egg counts [158]. These results were explained by the mutant *Na*-APR-1 ability to trigger neutralizing antibodies to both the native enzyme (*Na*-APR-1) and APR-1 orthologs from the other three human hookworm species [158]. *Na*-APR-1 (M74) administered to mice as low as 0.99 μg could trigger specific antibodies (IgG), which inhibited 89% of the native protease enzymatic activity, supporting this mutant protein as a vaccine candidate [159]. Additionally, serum from a rabbit vaccinated with *U. stenocephala* aspartic protease (*Us*-APR-1) showed cross-reactivity with an aspartic protease from a heterologous dog hookworm species, *A. ceylanicum* (*Ace*-APR-1) [67]. However, the possible APR-1 cross-protection between *A. ceylanicum* and *U. stenocephala* is still to be examined.

Hookworm GSTs are essential for parasite survival and offer promising targets for vaccine establishment. Recombinant *Ac*-GST-1 was highly immunogenic and could induce not only Th2-associated antibody (IgG1) and cytokine (IL-4) responses but also a strong Th1-like response indicated by the production of IFN-*γ* and IgG2 antibody response, suggesting *Ac*-GST-1 as a possible drug and vaccine target against hookworm infection [93]. Dogs vaccinated with *Ac*-GST-1 showed reduced worm burdens (39.4%) and egg counts (32.3%) but without statistical significance [93]. Additionally, hamsters immunized with *Ac*-GST-1 revealed significant worm reduction (50.6% and 53.7%) following the challenge with *N. americanus* larvae [28, 93]. In hamsters, r*Na*-GST-1 induced 32% and 39% drops in worm burdens [90]. Moreover, r*Na*-GST-1/Alhydrogel vaccine produced significant antigen-specific IgG responses, which were safe and well tolerated in both hookworm-naïve and exposed adults, stimulating this vaccine progress into children’s clinical trials [95]. The *Na*-GST-1/Alhydrogel vaccine has been proposed to act through the induction of IgG antibodies, that could be ingested by the hookworms developing inside the host intestine to block the detoxifying action of parasite *Na*-GST-1, thus allowing free heme to accumulate and damage the worm [95]. Recently, Brelsford et al. [160] have tested *Na*-GST-1/Alhydrogel vaccine immunogenicity and relative potency during storage. Although a 90% blockage of *Na*-GST-1 catalytic activity has been attained in animals vaccinated with stored *Na*-GST-1, a lower blockage percentage has been detected in immunized humans. Additionally, anti-*Na*-GST-1 antibodies obtained from volunteers from a hookworm non-endemic area exhibited more potent catalytic activity inhibition than those from volunteers from a hookworm-endemic area [160].

Some individual hookworm molecules with a possible role as immunomodulators were evaluated as vaccine candidates, such as *Ace-*KI and calreticulin. Recombinant *Ace*-KI-1 partially protected hamsters against the growth delay, with no detectable effect on anemia. These findings suggest that *Ace-*KI might contribute to hookworm-related malnourishment and growth delay pathogenesis by causing intestinal malabsorption [79]. Mice injected intraperitoneally with free calreticulin showed a 43–49% decrease in worm burdens in the lungs, with low serum IgE levels and intermediate levels of lung eosinophilia. However, calreticulin encapsulated in poly-lactide-coglycolide microparticles induced anti-calreticulin IgG1 and IgE levels and lung eosinophilia higher than that induced by free calreticulin but without protective immunity. These results indicate that calreticulin-protective immunity depends on an unknown mechanism other than the classical Th2 reaction, and alternative adjuvants should be explored [161].

Other ES products have been evaluated as vaccine candidates. Oral vaccination with *Ace*-ES-2 decreased anemia and improved the recovery rate in hamsters, implying that the parasite was neutralized by an anti-*Ace*-ES-2 immune reaction [125]. The oral *Ace*-FAR-1 vaccine significantly decreased the worm burden in hamsters, showing its possible function in hookworm biology and as a valuable vaccine target [144].

Some studies have suggested that combining multiple antigens may provide an effective vaccine development strategy to improve protection and/or disease symptoms in the host. *Ace*-ASP-2 and *Ace*-MTP-1 cocktail vaccine decreased worm burdens and egg counts and significantly enhanced Hb levels and body weight in hamsters compared with the single-antigen vaccine [162]. Currently, a divalent human hookworm vaccine consisted of *Na*-GST-1 and *Na*-APR-1 (M74) is under establishment by the Sabin Vaccine Institute Product Development Partnership. Both single antigen vaccines are in Phase 1 trials [163].

## Conclusions

Advances in molecular biology over the last 25 years resulted in the identification of numerous hookworm ES products, with diverse structures and functions. Many ES molecules help in host invasion, tissue migration, blood-feeding, nutrient acquisition, reproduction, embryo-development, host defense and immune modulation. Several hookworm ES products have been studied as potential vaccine and drug targets. Despite the tremendous progress in the study of hookworm ES products in recent decades, many key issues need to be further addressed. For example, the role of many molecules is still unclear, such as ASPs, *Ace*-ES-1, *Ace*-ES-2, *Ac*-KPI-1 and AChE. The substrate of *Ac-*API-1 needs to be determined. No studies have examined hookworm C-type lectin effects on host lectins and DC, larval exsheathment, and blood clotting. Similarly, the role of hookworm peroxiredoxins in immunomodulation remains to be analyzed. There are no available data about the cloning of SODs and AChE from hookworms. In addition, cDNA encoding calreticulin and C-TLs have not been cloned from *Ancylostoma* species. Proteomic analysis of ES products from adult *A. caninum* and *N. americanus* were previously conducted. However, proteomic analysis of ES products from other hookworm species and different life stages would uncover new molecules important in hookworm pathogenesis and vaccine development. Moreover, some molecules, with host defense and immunomodulatory roles, have not been tested as vaccine candidates, including TIMPs, MIF, NIF, SODs and PRXs. Targeting of molecules involved in blood-feeding, such as NAP and HPI, as vaccine candidates might prevent hookworm anemia. Although some hookworm ES products showed its efficacy as anti-inflammatory and antithrombotic agents, the hookworm protein therapy is yet to be fully explored.

## Data Availability

All data generated or analyzed during this study are included in the article.

## Abbreviations

ES: excretory/secretory
IDA: iron deficiency anemia
ASP: *Ancylostoma* secretory protein
ssL3: serum-stimulated infectious larvae
NAP: nematode anticoagulant peptide
HPI: hookworm platelet inhibitor
APR: aspartic protease
CP: cysteine protease
MTP: astacin-like zinc metalloprotease
KI/KPI: Kunitz-type protease inhibitor
TIL: trypsin inhibitor-like protein
API: aspartyl protease inhibitor
TIMP/TMP: tissue inhibitor of metalloprotease
MMP: matrix metalloprotease
GST: glutathione S-transferase
SOD: superoxide dismutase
PRX: peroxiredoxin
NIF: neutrophil inhibitory factor
MIF: macrophage migration inhibitory factor
C-TL/CTL: C-type lectin
AChE: acetylcholinesterase
FAR: fatty acid- and retinol-binding protein
NPA: nematode polyprotein antigen/allergen
